# Innate immunity stimulation effects on biofluid/imaging biomarkers and cognition in a non‐human primate model of sporadic AD pathology

**DOI:** 10.1002/alz70859_106012

**Published:** 2025-12-26

**Authors:** Noor Salaymeh, Akash G Patel, Pramod N Nehete, Bharti P Nehete, Ludovic Debure, Fengzhu Wang, Brianna Ryckman, Lisa M Pytka, Jody Swain, William D Hopkins, Michele Marie Mulholland, Thomas Wisniewski, Henrieta Scholtzova

**Affiliations:** ^1^ NYU Grossman School of Medicine, New York, NY USA; ^2^ UT MD Anderson Cancer Center, Michale E. Keeling Center for Comparative Medicine and Research, Bastrop, TX USA; ^3^ Small Animal Imaging Facility, UT MD Anderson Cancer Center, Houston, TX USA; ^4^ New York University Grossman School of Medicine, New York, NY USA

## Abstract

**Background:**

Non‐human primates (NHPs) are becoming a critical component of brain aging research, since many aspects of aging cannot be properly modeled in rodents. Our earlier published study utilizing a NHP model of sporadic AD‐related pathology that develops abundant CAA, the squirrel monkey (SQM), indicated that treatment with TLR9 agonist, CpG‐ODN 2006, safely ameliorates CAA while providing cognitive benefits. We advanced our research to evaluate the efficacy and safety of CpG‐ODN 1018, which has shown excellent safety profiles in clinical trials, including COVID vaccine trials and as an adjuvant in the FDA approved hepatitis B vaccine (HEPLISAV‐B/Dynavax). The present study was designed to assess CpG‐ODN immunostimulatory patterns and to monitor disease progression by a combination of behavioral measures and fluid/imaging biomarker signatures of pathology development.

**Method:**

Geriatric SQMs have received either CpG‐ODN or saline injections every 5 weeks. The efficacy of CpG‐ODN in triggering an immune response was analyzed using the Nanostring nCounter System. SIMOA and Luminex were used to measure plasma and CSF biomarkers of AD pathogenesis, neurodegeneration, and neuroinflammation. A touchscreen‐based Automated Cognitive Testing System (ACTS) was implemented within SQM cohorts to assess cognitive changes.

**Result:**

Longitudinal Nanostring analyses demonstrated significant upregulation of IFN‐inducible genes and specific cytokine‐chemokine genes (IFIT2, Mx2/MxB, GBP1, MIG, IP10, MCP1) following CpG‐ODN administration. Our preliminary analyses of plasma/CSF Ab40, Ab42 and Ab42/Ab40 ratio, NfL, GFAP, Neurogranin, and TREM2 levels did not reveal differences over time (post‐6th injection compared to baseline levels) or between our treatment groups. Subsequent characterization of biomarkers of inflammation and cerebrovascular dysfunction is underway. The potential of using ACTS to longitudinally monitor cognitive function in socially living SQMs was also demonstrated. Furthermore, no evidence of complications such as amyloid‐related imaging abnormalities (ARIA) was observed in our CpG‐ODN treated monkeys, confirming the safety of our immunomodulatory approach. We are expanding on these findings with longitudinal monitoring of biofluid/imaging biomarker profiles, as well as cognitive performance at selected intervals during the treatment period.

**Conclusion:**

Our comprehensive NHP study will further validate this concept of immunomodulation as a safer approach to effectively ameliorate dementia‐related pathology, particularly CAA, and support the potential clinical application of CpG‐ODN 1018.

